# Wnt signaling pathway-derived score for predicting therapeutic resistance and tumor microenvironment in lung adenocarcinoma

**DOI:** 10.3389/fphar.2022.1091018

**Published:** 2023-01-10

**Authors:** Hao-min Zhou, Li-mei Zhao

**Affiliations:** ^1^ Department of Intensive Care Unit, Shengjing Hospital of China Medical University, Shenyang, Liaoning, China; ^2^ Department of Pharmacy, Shengjing Hospital of China Medical University, Shenyang, Liaoning, China

**Keywords:** lung adenocarcinoma, tumor microenvironment, immunotherapy targets, unsupervised clustering, molecular target

## Abstract

**Background:** Lung adenocarcinoma (LUAD) is the most common subtype of lung cancer. Due to tumor heterogeneity, understanding the pathological mechanism of tumor progression helps to improve the diagnosis process and clinical treatment strategies of LUAD patients.

**Methods:** The transcriptome pattern, mutant expression and complete clinical information were obtained from the cancer genome atlas (TCGA) database and microarray data from gene expression omnibus (GEO) database. Firstly, we used single sample Gene Set Enrichment Analysis (ssGSEA) to estimate the activation of Wnt signaling pathway in each sample. Consensus clustering algorithm was used to classify LUAD samples into different subgroups according to the transcription patterns of 152 Wnt signaling pathway related genes. Then, ESTIMATE, ssGSEA and Gene Set Variation Analysis (GSVA) algorithms were used to assess the biological pathways and immunocytes infiltration between different subtypes. LASSO-COX algorithm was conducted to construct prognostic model. Kaplan-Meier and multivariate Cox analysis were performed to evaluate the predictive performance of risk model. Gene features were further confirmed using external datasets. Finally, we conducted vitro assay for validating hub gene (LEF1).

**Results:** Based on the transcription patterns of 152 Wnt signaling pathway related genes, four different subtypes of LUAD patients were screened out by consensus clustering algorithm. Subsequently, it was found that patients with cluster A and B had massive immunocytes infiltration, and the survival rate of patients with cluster B was better than that of other subgroups. According to the coefficients in the LASSO- Cox model and the transcriptome patterns of these 18 genes, the risk score was constructed for each sample. The degree of malignancy of LUAD patients with high-risk subgroup was remarkable higher than that of patients with low-risk subgroup (*p* < 0.001). Subsequently, five top prognostic genes (AXIN1, CTNNB1, LEF1, FZD2, FZD4.) were screened, and their expression values were different between cancer and normal tissues. FZD2 and LEF1 were negatively related to ImmunoScore, and AXIN1 was negatively related to ImmunoScore. The significant correlation between LUAD patient risk score and overall survival (OS) was verified in external datasets. In the A549 cell line, knockdown of LEF1 can reduce the invasive and proliferation ability of LUAD cells.

**Conclusion:** A innovative 18 genes predictive feature based on transcriptome pattern was found in patients with lung adenocarcinoma. These investigations further promote the insight of the prognosis of lung adenocarcinoma and may contribute to disease management at risk stratification.

## Introduction

Lung adenocarcinoma (LUAD) is the main cause of cancer-related deaths worldwide, accounting for about 40% of lung cancer patients (Nguyen et al., 2022). Over the past decade, treatment for epidermal growth factor receptors and anaplastic lymphoma kinases has benefited only a small proportion of LUAD patients (Wang et al., 2019; Wang et al., 2020). Clinically defined LUAD molecular subtypes urgently need precise treatment.

Previous studies have integrated different data types such as transcriptome, genome, and metabonomics to characterize the molecular mechanism of lung cancer and predict the survival status of cancer patients. Zou et al. (2020) quantified the infiltration of immunocytes in 32 types of cancer and observed considerable heterogeneity in the prognostic correlation of these immunocytes in different types of cancer. An immune cell feature score model with good prognosis was constructed for LUAD, which can further deepen our understanding of the LUAD and have an impact on immunotherapy (Zuo et al., 2020). On the basis of co-occurrence of KEAP1 mutation, Marinelli et al. (2020) identified four genes that may be related to the reduction of immunotherapy effect (KEAP1, PBRM1, SMARCA4, and STK11). This study suggested that co-existing changes in a limited set of genes were the characteristics of LUAD patients who are no response to immunotherapy and high TMB. The immune cold microenvironment may explain the clinical process of the disease. Similar to other cancers (Troiano et al., 2020), LUAD also exhibits peculiar molecular and clinical behavior compared to lung squamous cell carcinoma.

Recently, studies have been conducted to generate genetic signatures that predict prognostic risk in patients with lung adenocarcinoma. Zhang et al. (2020b) collected seven cohorts’ of 1300 patients with LUAD and constructed the first tumor Necrosis Factor family-based model to predict the clinical outcome and immune status of LUAD patients (Zhang et al., 2020a). Zhao et al. (2020) obtained RNA sequencing data of LUAD from the cancer genome atlas (TCGA) database, as well as microarray data from the gene expression omnibus (GEO) database, and found a new type of 19 prognostic characteristics based on transcriptome pattern in LUAD patients. Compared with patients with high-risk scores, the mortality risk of patients with low-risk scores was reduced by 81%. The above investigation identified different genetic characteristics for prognostic risk prediction by using different algorism and presented different genomics profile.

Wnt signaling pathway is a classic tumor activation pathway related to tumor progression, which regulates cell growth, differentiation and migration. (Yang et al., 2017; Pan et al., 2020; Li et al., 2021a). There are three Wnt signaling pathways that have been described so far: classical β-catenin dependent pathway, non-classical Wnt/calcium pathway and non-classical planar cell polarity pathway (Zhao et al., 2019; Abplanalp et al., 2021). Wnt signal abnormalities are associated with some tumor disease, and the most significant ones are lung cancer, breast cancer, bladder cancer, clear cell renal cell cancer and prostate cancer (Xu et al., 2022; Xue et al., 2022; Zhao et al., 2022). Most investigation on Wnt signaling pathway in lung adenocarcinoma only focus on Wnt pathway as a downstream signaling pathway to regulate the proliferation and differentiation of lung adenocarcinoma cells (Tammela et al., 2017; Wu et al., 2021).

This investigation did not demonstrate the effect of Wnt signaling pathway as a whole regulatory profile on the results of lung adenocarcinoma. Therefore, current studies have identified the correlation between Wnt signaling pathway and clinicopathologic parameters of cancer by using transcriptome pattern downloaded from TCGA website, and identified the impact of Wnt pathway-related genes on lung adenocarcinoma results. Moreover, a predictive model was established based on transcriptome pattern, and its applicability and predictive performance in lung adenocarcinoma were evaluated.

## Materials and methods

### Dataset collecting and processing

The transcriptome pattern, mutant expression and complete clinical information were obtained from the TCGA website and microarray data from GEO website. Patients without complete clinical data were excluded from assessment. A total of two datasets (GSE68465 and GSE72094) were downloaded, and the combat method of “sva” R package was employed to remove the batch effect. The merged TCGA and GEO cohort were named as meta-cohort.

### Consensus clustering of lung adenocarcinoma

We first collected 152 Wnt signaling pathway related genes from previous articles (Sun et al., 2021). According to the transcriptome pattern of 152 Wnt signaling pathway-related genes, the optimal k-means clustering (“kmeans” function in R tool) was employed to assign different distribution information to each patient, and each patient was classified into our subgroups. The “ConsensusClusterPlus” R package was employed for cluster analysis, and 1000 cycles were calculated to ensure stability and reliability. Kaplan-Meier algorism was performed to assess the total survival (OS) rate between different subgroups.

### Identification of differential pathways

The GSVA algorithm was conducted to assess the differences in biological pathways between subgroups. GSVA algorism mainly evaluated gene set enrichment based on microarray nuclear transcriptome level. The principle of GSVA algorithm is to calculates the expression matrix of gene set among samples, so as to evaluate whether the mechanism pathway is enriched among different samples (Hänzelmann et al., 2013). c2. cp. kegg. v7.0. symbols as a reference gene set, FDR <0.05 as a screening threshold.

### Comparison of immunocytes infiltration

In order to investigate the component of immunocytes infiltration between the four subtypes, we employed the “ESTIMATE” technological method to estimate immune score and stromal score for further prediction of immunoreaction and tumor microenvironment. Then we conducted the ssGSEA technological method to assess the enrichment level of immunocytes infiltration and immune-related pathways based on meta-cohort. Mann-Whitney U test was performed to compare the differences among the four subgroups.

### LASSO-COX regression and prognostic signature verification

In order to improve the predictive performance and feasibility of the Wnt signal pathway related model, Lasso-Cox model was conducted to analyze the correlation between the clinical characteristics of Wnt signaling pathway and the risk score. Using “glmnet” R package, the best Wnt signaling pathway genes were screened and the prognosis model was constructed. Use the following formula to generate risk score:

Risk score = expression*gene_i_ × coefficient*gene _i_

Importantly, we used coefficient of each gene in multivariate cox regression. The according to the median risk, the patients were divided into high-risk group and low-risk group. Target genes in the model include risk genes and protective genes. HR > 1 was considered as risk factor, whereas it was a protective factor. Then, Kaplan-Meier survival model and ROC curve model were draw to assess the predictive performance of the model. We input the risk signature genes into the STRING database and employed MCC method to identify the key Top 5 molecular in the protein-protein interaction network.

### Vitro assay

The shRNAs targeting LEF1 were designated by Biomics Biotechnologies Co. Ltd. (Nantong, China). The expression plasmids, pU6H1-GFP-shLEF1-1, -2, -3, and controls were constructed. Sequences of targeting LEF1 from references as follows (Xiao et al., 2021): shRNA1: GCG​ATT​TAG​CTG​ACA​TCA​A, shRNA2: AGA​TGT​CAA​CTC​CAA​ACA​A, shRNA3: GTT​GCT​GAG​TGT​ACT​CTA​A, shRNA-NC: TTC​TCC​GAA​CGT​GTC​ACG​T. Additional experimental details are presented in our previous study (Feng et al., 2022a; Feng et al., 2022b; Cheng et al., 2022).

## Results

### Wnt signaling score based on ssGSEA in the multicenter study

**FIGURE 1:**
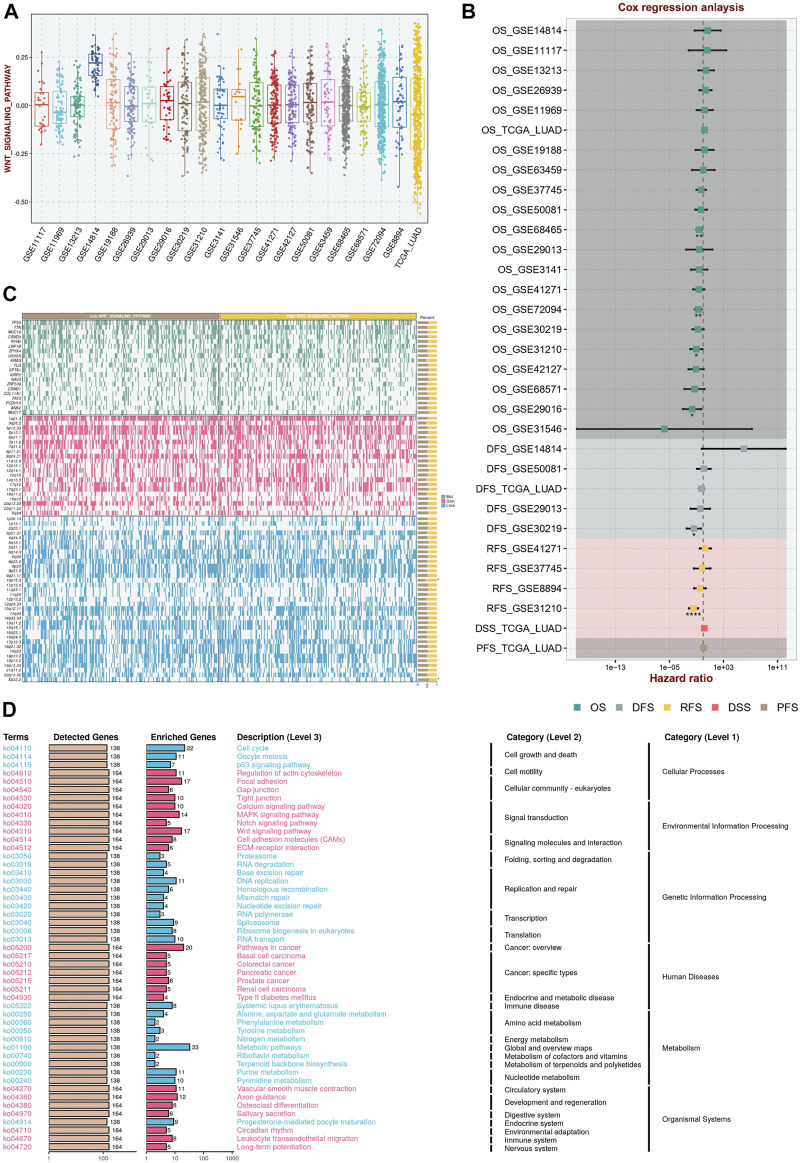
Wnt Signaling Score Based on ssGSEA in the Multicenter Study. **(A)** The Wnt signaling pathway absolute enrichment score based ssGSEA in different datasets. **(B)** Cox regression analysis in multicenter study demonstrated that Wnt scores had prognostic value. **(C)** The mutational landscape in different Wnt groupings. **(D)** The activation pathways in different Wnt groupings.

The Wnt signaling pathway absolute enrichment score were calculated in each dataset (Figure 1A). The range of Wnt scores was approximated in most of the datasets except for GSE14814 dataset. In addition, cox regression analysis demonstrated that Wnt scores had prognostic value in the GSE31210, GSE30129, GSE29016, GSE31210, GSE72094 and GSE68465 datasets (Figure 1B). Interestingly, the mutational landscape (Figure 1C) as well as the activation pathways (Figure 1D) were significantly different in different Wnt groupings.

### Relationship between Wnt signaling pathway related genes and phenotypic characteristics of lung adenocarcinoma

**FIGURE 2:**
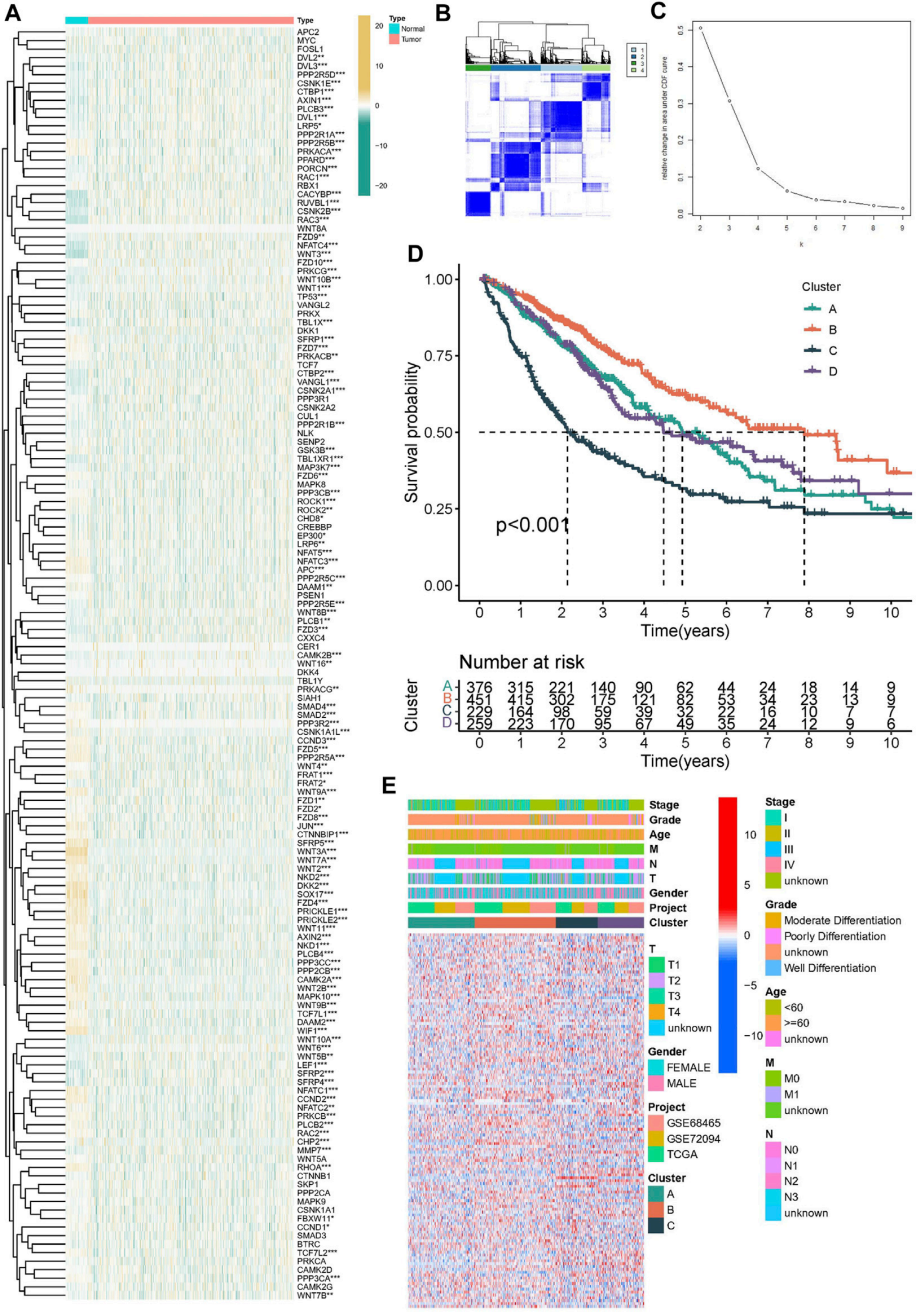
Construction of molecular subtypes in lung adenocarcinoma. **(A)** The heatmap was utilized to present the transcriptome pattern of 152 wtn signal pathway related genes in distinct samples. **(B,C)** Unsupervised clustering was conducted to divided samples into four subgroups performed according to the 152 wtn signal pathway related genes patterns. **(D)** Kaplan–Meier survival model for lung adenocarcinoma, *p* < 0.001. **(E)** The heatmap was utilized to present the transcriptome pattern of 152 wtn signal pathway related genes in distinct subtypes. Red/blue represented high/low expression of genes. The comments on the right include TNM stage, clinical grade/stage, gender, age, project and cluster, respectively.

In order to explore whether Wnt signaling pathway related genes play a regulatory role in lung adenocarcinoma. We first explored the Wnt signaling pathway related genes in cancer tissues and normal tissues based on TCGA dataset. Heatmap showed the expression level of Wnt signaling pathway related genes (Figure 2A). We found that there were significant differences in molecular expression between normal tissues and tumor tissues. Based on the expression pattern of Wnt signaling pathway related genes, we classified the lung adenocarcinoma patients into different subgroups. Using the similarity of Wnt-related gene expression, we selected the value of k = 4 (Figures 2B,C). The lung adenocarcinoma patients from meta-cohort dataset were divided into four subgroups (Cluster A, Cluster B, Cluster C and Cluster D). The four subgroups contained 376 samples, 451 samples, 229 samples and 259 samples, respectively. As shown in Figure 2D, K-M survival curve analysis showed that cluster B had the best clinical outcomes, and cluster C had the shortest survival time (*p* < 0.001). We compared the expression patterns of Wnt signaling pathway-related genes in these subgroups (Figure 2E).

### Differences of TME infiltration among four subtypes of Wnt signaling pathway

**FIGURE 3:**
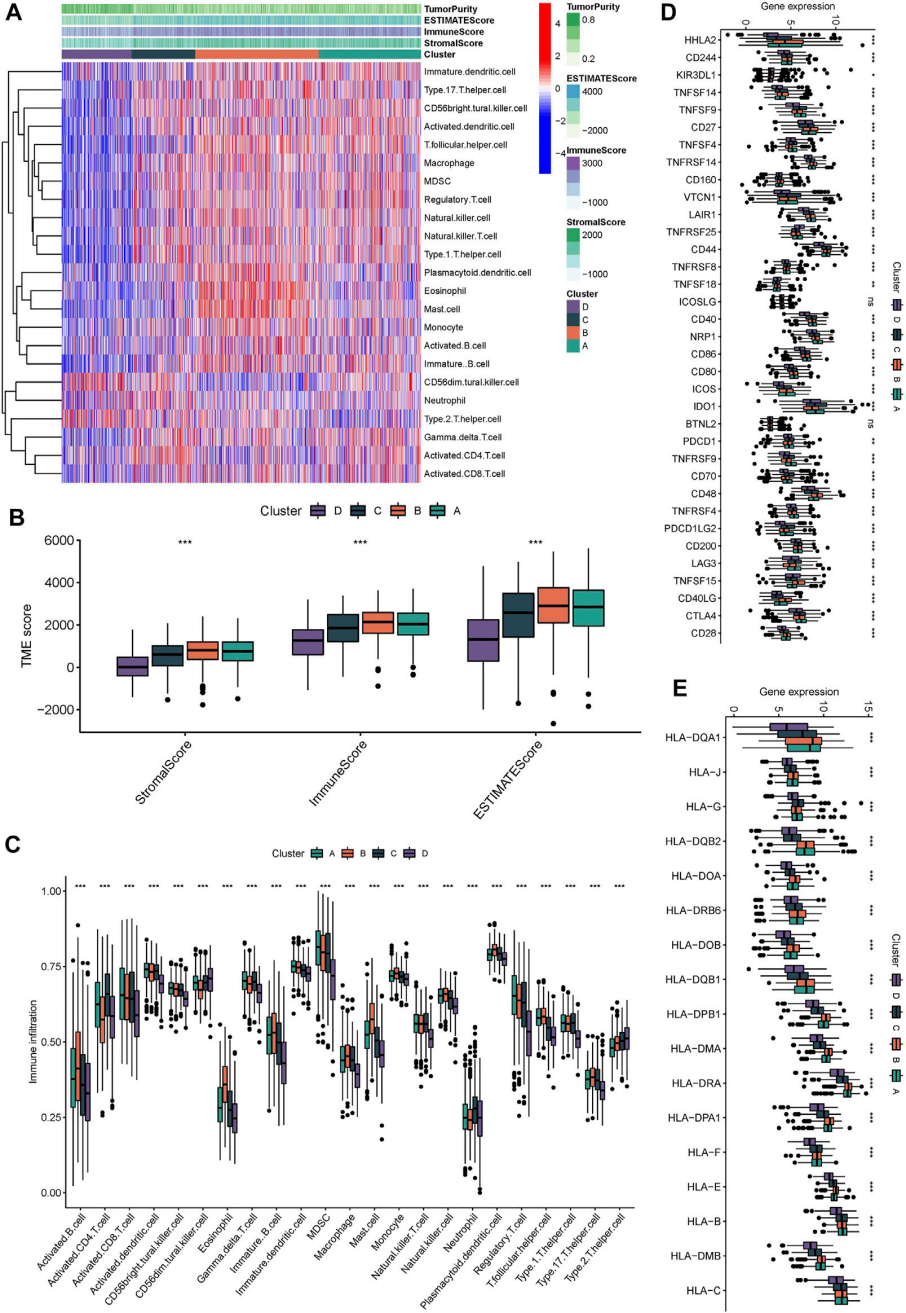
Immunological characteristic of different molecular subgroups. **(A)** the heatmap displayed the immunocytes infiltration in different molecular subgroups. **(B)** the stromalScore, immunoScore, ESTIMATEScore was calculated by “ESTIMATE” algorism. **(C)** the immunocytes infiltration in different molecular subgroups. **(D,E)** Expression levels of immunomodulators in different molecular subgroup, The median value: black lines in boxes, the outliers: black dots out boxes.

Subsequently, we explored the tumor immune microenvironment among different subtypes to attempt to explain that Wnt signaling pathway affects the clinical outcome of patients by regulating the tumor immune microenvironment. We analyzed the information of immune cell infiltration and found that activated innate immune cells in cluster A and B were abundant, including activated dendritic cells, CD56dim natural killer cells, macrophages, activated B cells, and activated CD4 and CD8 T cells, which had significant survival advantages (Figure 3A). Although some tumor tissues have a large number of immune cells, these immune cells cannot penetrate the tumor and are forced to remain in the surrounding stromal tissues. Therefore, stromal activation in tumor microenvironment is considered to be immunosuppressive (333). Therefore, the clinical outcome of Cluster A is not ideal. In this investigation, the “ESTIMATE” algorism also showed that the immune scores of cluster A and B were higher than those of cluster C and D (Figure 3B). We also noticed that although the types of immunocytes infiltration were consistent in different subtypes, the proportion of immunocytes in different subgroups was different. This indicates that tumor regulatory pathways such as Wnt, RAS and other signaling pathways do not alter the types of immune infiltrating cells, but they may change their proportions. Figure 3C verified the above results. Subsequently, we continued to detect the expression levels of immunomodulators among different subtypes. The Mann-Whitney U test was conducted to compare differences between the four subgroups. Immunomodulators such as CD27, CD86, CTLA4 and HLA family were highly expressed in cluster A and B. Immunomodulators induce immune response and regulate immune response. This further confirmed that cluster A and B have higher immune cell infiltration levels than cluster C and D (Figures 3D,E).

### Identification of biological behavior patterns

**FIGURE 4:**
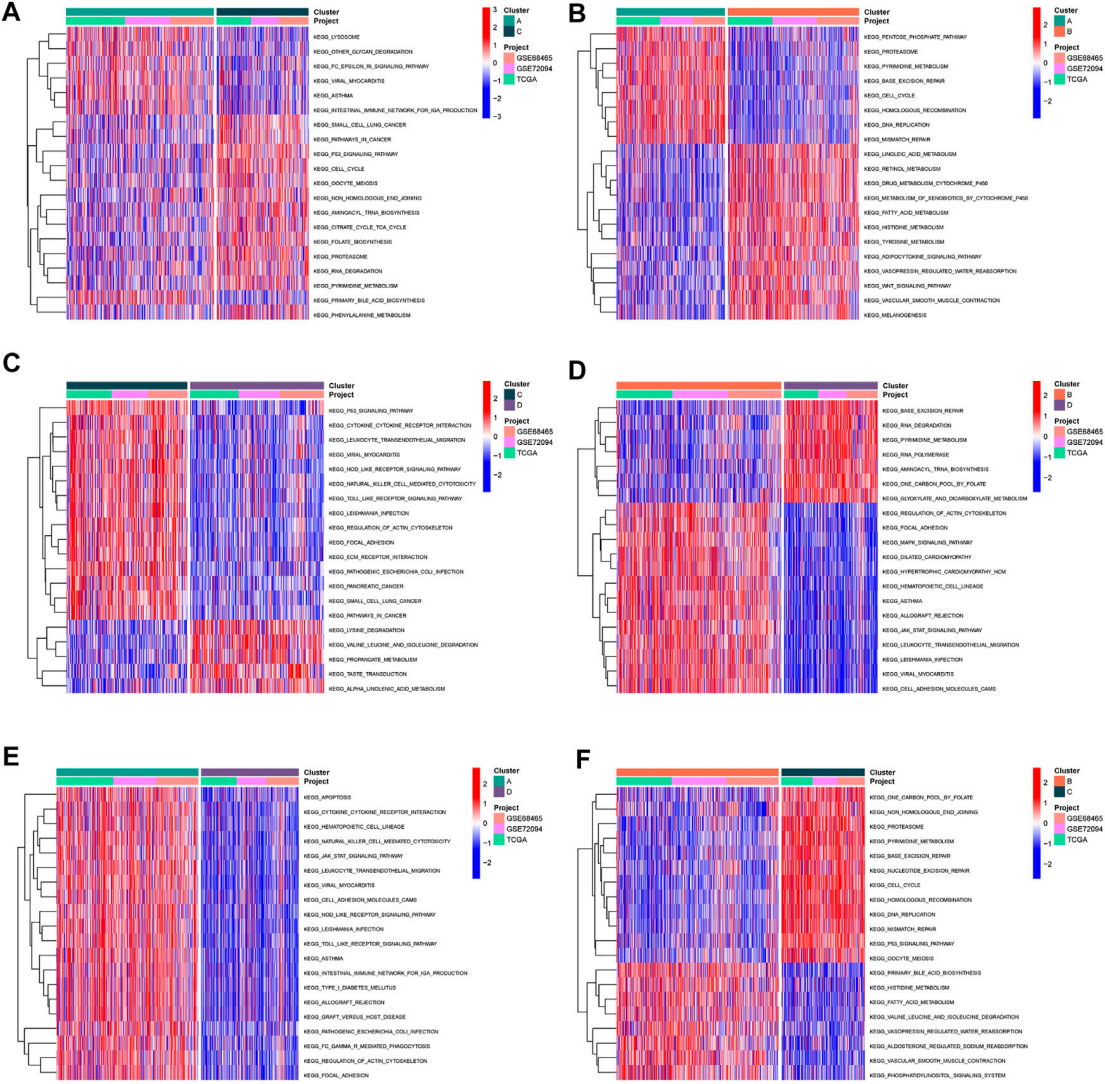
Pathway enrichment analysis **(A–F)** The heatmap displayed the biological processes among each cluster, plotted by GSVA algorithm.

Subsequently, we conducted GSVA enrichment analysis to identify the differences in biological behaviors among these four subgroups, so as to determine the pathways through which Wnt signaling pathway-related genes regulate malignant differentiation of tumor cells. Cluster A presented with Cell cycle, DNA replication, mismatch repair. Cluster B showed enrichment of metabolic pathways, including linoleic acid metabolism, retinal metabolism, drug metabolism cytochrome P450 signaling pathways. Cluster C shows enrichment of carcinogenic activation pathways, such as small cell lung cancer, cell cycle, pathway in cancer, and P53 signature. Cluster D is enriched in RNA degradation, base excision repair and other signaling pathways (Figure 4).

### Prognostic prediction model of Wnt pathway in lung adenocarcinoma

**FIGURE 5:**
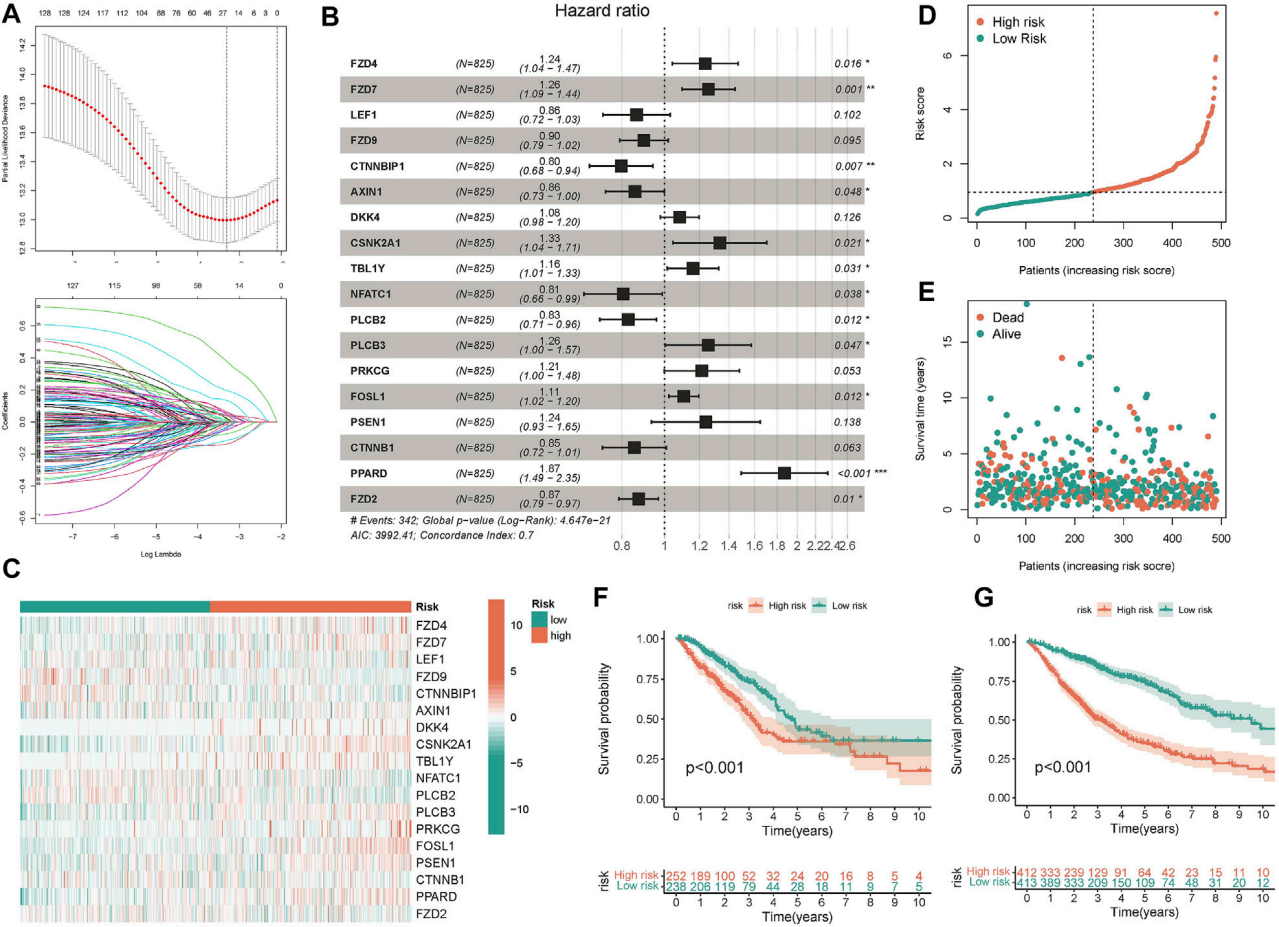
Construction and validation of predictive model. **(A)** 26 candidate genes were selected by LASSO regression **(B)** The forest map displayed the hazard ratios of the 18 target genes **(C)** The heatmap displayed the expression values of 18 target genes in different risk score groups. **(D,E)** The scatter plot depicts the distribution of patient risk values and mortality. **(F,G)** The K-M survival model was constructed to explore the predictive performance.

**FIGURE 6:**
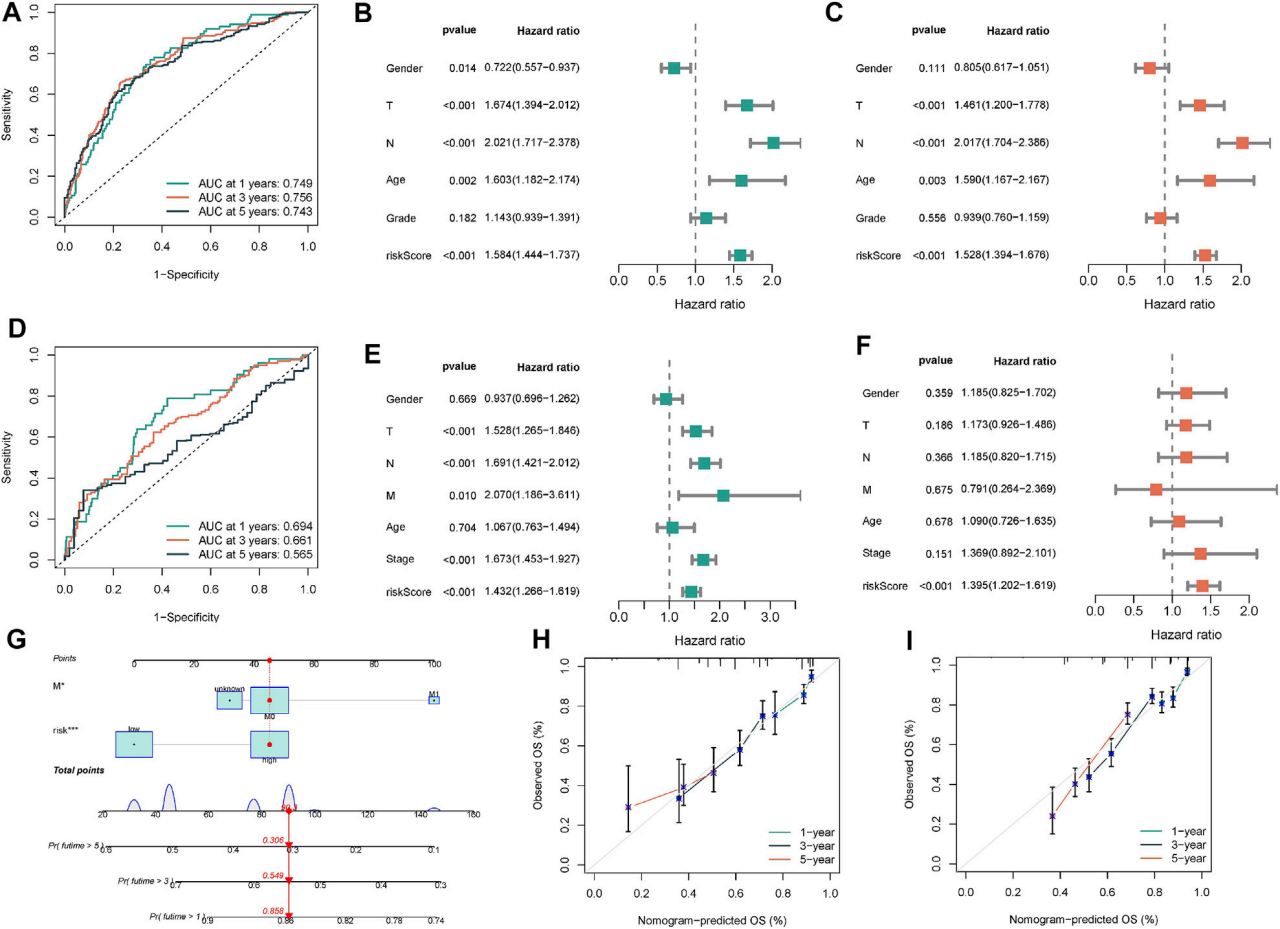
Construction and validation of nomogram. **(A)** The ROC curve was performed to verify the predictive performance of predictive model. **(B,C)** Univariate/multivariate Cox regression model was constructed to verify the independent predictive performance of each parameter in TCGA-BLCA cohort. **(D–F)** ROC curve and Univariate/multivariate Cox regression model were plotted to verify in GEO database. **(G)** Construction of a nomogram combining M stage and risk score. **(H,I)** Calibration plot showing that nomogram-predicted survival probabilities corresponded closely to the observed proportions.

After discovering that Wnt signaling pathway was related to the clinical results of lung cancer, we tried to establish prognostic risk score based on Wnt signaling pathway. We collected 152 Wnt pathway related genes. After applying the LASSO algorithm to these 152 genes, we removed highly correlated genes and reduced the dimension of subsequent multivariate COX algorithm (Figure 5A). Subsequently, COX multivariate model was constructed to screen the final prognostic genes. A total of 18 genes were identified as independent prognostic genes, including FZD4, FZD7, LEF1, FZD9, CTNNBIP1, AXIN1, DKK4, CSNK2A1, TBL1Y, NFATC1, PLCB2, PLCB3, PRKCG, FOSL1, PSEN1, CTNNB1, PPARD, and FZD2. The LASSO-Cox regression coefficient was integrated with the corresponding gene expression values to establish a risk marker feature (Figure 5B). According to the median risk marker, we divided lung adenocarcinoma patients into high-risk group and low risk group. The heatmap showed the transcriptome pattern of prognostic genes in high and low risk patients. Red represents high expression, while green represents low expression (Figure 5C). The scatter plot displayed the risk score of patients, and the correlation between mortality and risk score. With the increase of risk value, mortality increases (Figures 5D,E). In addition, the K-M survival curve showed that the overall survival time of the low-risk group was longer than that of the high-risk group in TCGA cohort and GEO cohort (Figures 5F,G). In order to test the predictive performance of the prognostic model, we draw the ROC curve, and the area under the curve (AUC) was used to evaluate the 1, 3, and 5–year survival rates in TCGA cohort, 0.749, 0.756, and 0.743 respectively (Figure 6A). The multivariate Cox model proved that the prognostic model can independently predict the clinical outcomes of lung adenocarcinoma patients (Figures 6B,C). As an external validation, we performed ROC curve, univariate and multivariate Cox model analysis again. The area under the curve (AUC) was used to evaluate the 1, 3 and 5–year survival rates in GEO cohort, 0.694, 0.661, and 0.565 respectively (Figure 6D). The results of univariate and multivariate Cox analysis were consistent with the above (Figures 6E, F). Clinical pathological parameters, especially TNM staging, are closely related to the prognosis of patients. In order to improve the accuracy of the prediction model, we integrated M stage and risk score to construct a nomogram for improving the prediction performance (Figure 6G). The calibration curve of the established nomogram showed high accuracy between the actual observation value and the predicted value (Figures 6H, I).

### Association of five genes with immune microenvironment in lung adenocarcinoma

**FIGURE 7:**
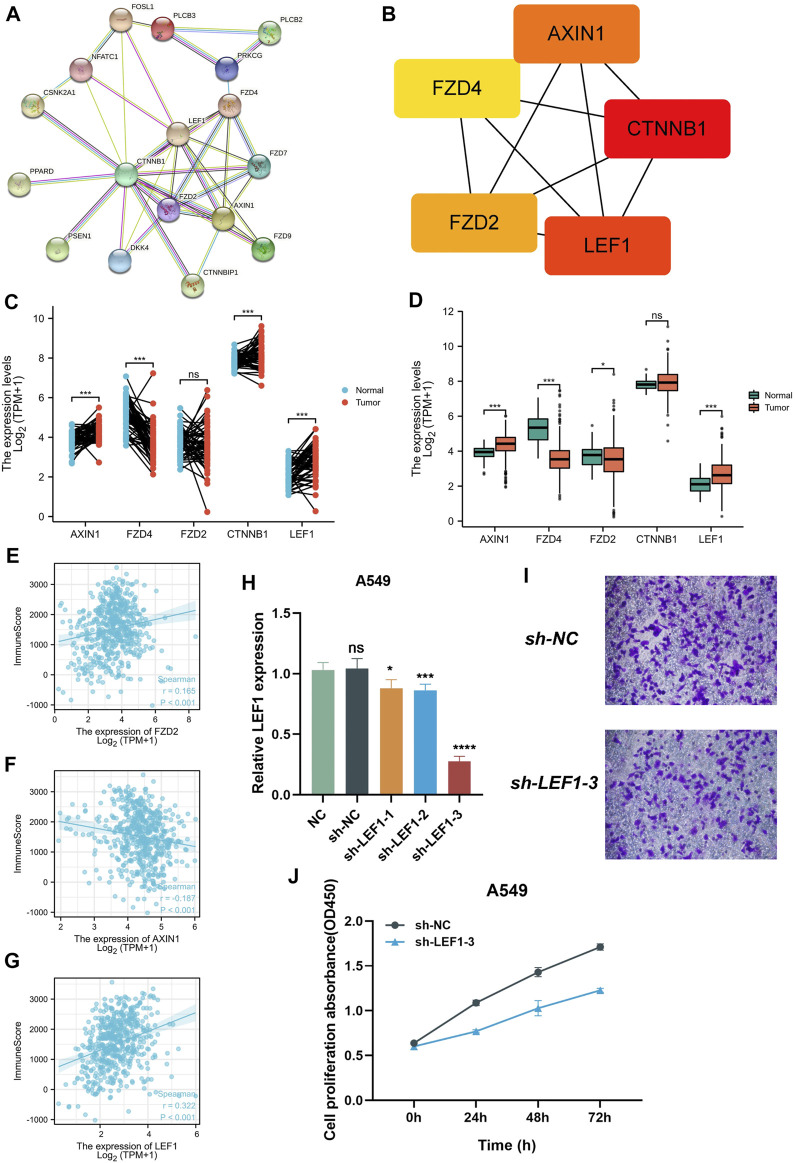
Collection of key prognostic genes. **(A)** Protein-protein interaction network diagram of 18 target genes **(B)** MCC method was performed to identify the key Top 5 molecular in the protein-protein Interaction network. **(C,D)** the expression level of 5 key prognostic genes in normal tissues and tumor tissues **(E–G)** the Correlation between 5 key prognostic genes and immune scores. **(H)** qPCR assay detect the expression of LEF1. **(I)** Transwell assay detect the invasion of the A549 cell line after knockdown LEF1. **(J)** CCK-8 assay detect the proliferation of the A549 cell line after knockdown LEF1.

In order to explore the mechanism of 18 prognostic genes, we constructed the interaction diagram of 18 prognostic genes (Figure 7A). MCC method was used to identify the key Top 5 proteins in the protein-protein interaction network, including AXIN1, CTNNB1, LEF1, FZD2, FZD4 (Figure 7B). Therefore, we speculate that the above five top genes may be the key molecules affecting the disease progression. We detected the expression level of these molecule in normal tissues and cancer tissues of lung adenocarcinoma patients in TCGA and GEO cohorts. We found that AXIN1, CTNNB1 and LEF1 were highly expressed in tumor tissues and FZD4 was highly expressed in normal tissues in TCGA cohort (Figure 7C). In GEO cohort, AXIN1 and LEF1 were highly expressed in tumor tissues, while FZD4 and FZD2 were highly expressed in normal tissues (Figure 7D). Subsequently, we detected the correlation between these five genes and immune scores. FZD2 and LEF1 were positively related to immune scores, and AXIN1 was negatively related to immune score (Figures 7E–G). Finally, we conducted vitro assay for validating the carcinogenic potency of LEF1 (Figures 7H–J). In the A549 cell line, knockdown of LEF1 can reduce the invasive and proliferation ability of LUAD cells.

## Discussion

Lung adenocarcinoma is the most common form of lung cancer and the deadest cancer in the world. In order to characterize the genomic and transcriptome abnormalities of lung adenocarcinoma and identify the survival outcomes of patients, different data types such as transcriptome, genome, and metabonomics have been employed for comprehensive analysis (Jin et al., 2020; Sun et al., 2020). Recently, prognostic risk features have emerged to cluster lung adenocarcinoma patients. In our investigation, we used RNA-seq data which downloaded from the TCGA website to determine the correlation between Wnt signaling pathway and the clinical outcomes of lung adenocarcinoma. In addition, a prognostic model was established based on transcriptome pattern, and its applicability and value in lung adenocarcinoma were evaluated.

Abnormalities of Wnt signaling pathway is usually associated with cancer (Lei et al., 2021). The characteristics of lung adenocarcinoma include abnormal epigenetic regulation of Wnt pathway genes and inactivation of tumor suppressor genes (Hou et al., 2021). Tumor suppressor genes inhibit Wnt signaling pathway. However, the silencing of these tumor suppressor genes leads to the activation of Wnt signaling pathway, which is involved in the occurrence or progression of human malignant tumors (Líbalová et al., 2014). NKX2-1/ERK drived Wnt pathway to promote cell proliferation, shorten the value-added cycle, and increased the malignancy of lung adenocarcinoma. BRAF/MEK inhibitors drived NKX2-1 positive tumor cells into a stationary state, while NKX2-1 negative cells cannot exit the cell cycle after the same treatment. These data clarify the complex interrelationship between lineage specificity and carcinogenic signaling pathways, which may affect lineage-specific treatment strategies in regulating lung adenocarcinoma characteristics. Kerdidani et al. demonstrated that Wnt silenced chemokine genes in dendritic cells and induces adaptive immune resistance in lung adenocarcinoma. Moreover, we examined whether 18 target genes from classical and non-classical Wnt signaling pathways could accurately detect the risk of lung adenocarcinoma. The report of the Guidelines for Prognosis of Tumor Markers has recently been applied in many journals (Zhang et al., 2020b).

Based on the TCGA data, we constructed a lung adenocarcinoma prognosis model, and we found five key Wnt signaling pathway related genes, including AXIN1, CTNNB1, LEF1, FZD2, FZD4. These key genes were related to cancer and play an indispensable role in lung adenocarcinoma pathway. These markers were candidate genes for molecular targeting. Li et al. (2021b) confirmed that AXIN1 encoding the negative regulator of Wnt/β-catenin signal was the direct target of YTHDF2. YTHDF2 promotes AXIN1 mRNA decay and subsequently activates Wnt/β-catenin signal. Knockout of AXIN1 fully rescued the inhibitory effect of YTHDF2 depletion on proliferation, colony formation and migration of lung cancer cells. These results suggest that YTHDF2 promotes the development of LUAD by upregulating AXIN1/Wnt/β-catenin signaling, which may be a potential therapeutic target for LUAD. Zhou et al. 2022 collected 564 patients with lung adenocarcinoma, of which 30 (5.3%) carried CTNNB1 mutations. The study found that female patients and non-smokers had high CTNNB1 mutations, and the clinical outcomes of primary lung adenocarcinoma with CTNNB1 mutations was poor (Zhou et al., 2019). Nguyen et found that LEF1 and HOXB9 mediated Wnt/TCF signal transduction to promote lung adenocarcinoma metastasis, indicating that LEF1 may be a target for lung adenocarcinoma metastasis (Nguyen et al., 2009). In addition, Li et al. 2021a collected clinical information of LUSC patients from the Cancer Genome Atlas database and related methylation sequences from the University of California, Santa Cruz database to construct methylation subtypes and analyze clinical outcomes. The researchers constructed a predictive model based on the difference of DNA methylation level to classify the molecular subtypes of LUSC patients, and provided more personalized clinical treatment strategies according to different clinical subtypes. GNAS, FZD2 and FZD10 are the three core genes that may be related to the prognosis of LUSC patients (Li et al., 2021c).

First of all, our investigation was worthy of recognition, which obtained Wnt-related prognostic genes, and then constructed a Wnt-related scoring model. The function of Wnt is most common in embryogenesis and tumorigenesis, but previous literature has only described the carcinogenic effect of Wnt and rarely investigated its predictive ability. Therefore, our study opened up a new horizon for exploring the Wnt pathway. Our research has some limitations. First of all, our investigation was based on the public database for multi-omics analysis, which was a retrospective study with subjective bias. Future investigation needed to validate the diagnostic value of 18 candidate genes in fresh frozen biopsy or peripheral blood. Secondly, our research had a moderate sample size and needed a larger case-control study for LUAD patients.

## Data Availability

The original contributions presented in the study are included in the article/supplementary material, further inquiries can be directed to the corresponding author.
